# A Particular Use of Endobag^®^: Extraction of Rectal Foreign Bodies

**DOI:** 10.1155/2017/8909706

**Published:** 2017-12-20

**Authors:** G. Terrosu, V. Cherchi, S. Calandra, A. Esposito, M. Marino, D. Berretti, A. Risaliti

**Affiliations:** ^1^General Surgery and Transplantation Unit, Department of Medical and Biological Sciences, University of Udine, Udine, Italy; ^2^Department of Gastroenterology and Digestive Endoscopy, Academic Center “Santa Maria della Misericordia”, Udine, Italy

## Abstract

**Introduction:**

The incidence of foreign bodies (FBs) in the rectum has recently increased. FB removal by the transanal way or by colonoscopy is generally feasible and only in few cases surgery is strictly necessary. Due to FB dimensions or rectum and colon anatomy, sometimes it may represent a challenge.

**Materials and Methods:**

Two cases of FB inserted in the rectum were treated in our institute. They underwent surgery using Endobag, a laparoscopic surgical device. The device was inserted through the anus in order to catch and remove the FB.

**Results:**

Both the procedures were easily performed, without any complication.

**Conclusions:**

The use of Endobag seems to be a good and effective way to remove FB from rectum.

## 1. Introduction

The presence of foreign bodies (FBs) in the rectum is encountered as an uncommon finding in the Emergency Departments, although the incidence has recently increased due to the modification of the sexual behaviours and the use of various objects for sexual purposes, mainly introduced by patients for self-erotism [1]. Between 2009 and 2011, a total of 3359 hospitalizations with the primary diagnosis of rectal foreign body have been recorded in USA [2]. In particular, a bimodal age distribution has been observed: patients in their twenties had FBs for anal erotism or because of sexual assaults, while patients in their sixties mainly used them for prostatic massages or to break faecalomas. A useful classification of rectal foreign bodies categorizes them as voluntary versus involuntary and sexual versus nonsexual [3]. Less frequently, FB presence is due to iatrogenic causes such as migration of medical devices in the intestinal lumen [4, 5]. In rare cases, the FB consists of objects stuck in the colon after ingestion [6].

The management of patients with FB in the rectum is often very challenging due to the different characteristics of retained objects. After rectal lesions have been excluded by radiological exams, a transanal extraction is required. Although it is usually quite easy to remove the FB at the bedside, sometimes removal of the object requires a more invasive procedure, often performed in the operation theatre under proper anesthesia, in some cases with the need of a laparoscopic approach. Laparotomy for retrieval should only be used as a last resort after failure of attempts at transanal removal, presence of perforation, and/or peritonitis [7]. Many procedures have been proposed for removal of retained FBs: there is not a standardized procedure for this purpose because of different shapes, sizes, and materials of the retained objects. For this reason, physicians often have to use their imagination to find the best technique to apply.

Here, we describe two cases with rectal retained FB treated at our institute, that required the use of a laparoscopic surgical device.

## 2. Materials and Methods

### 2.1. Case 1

**Figure 1:**
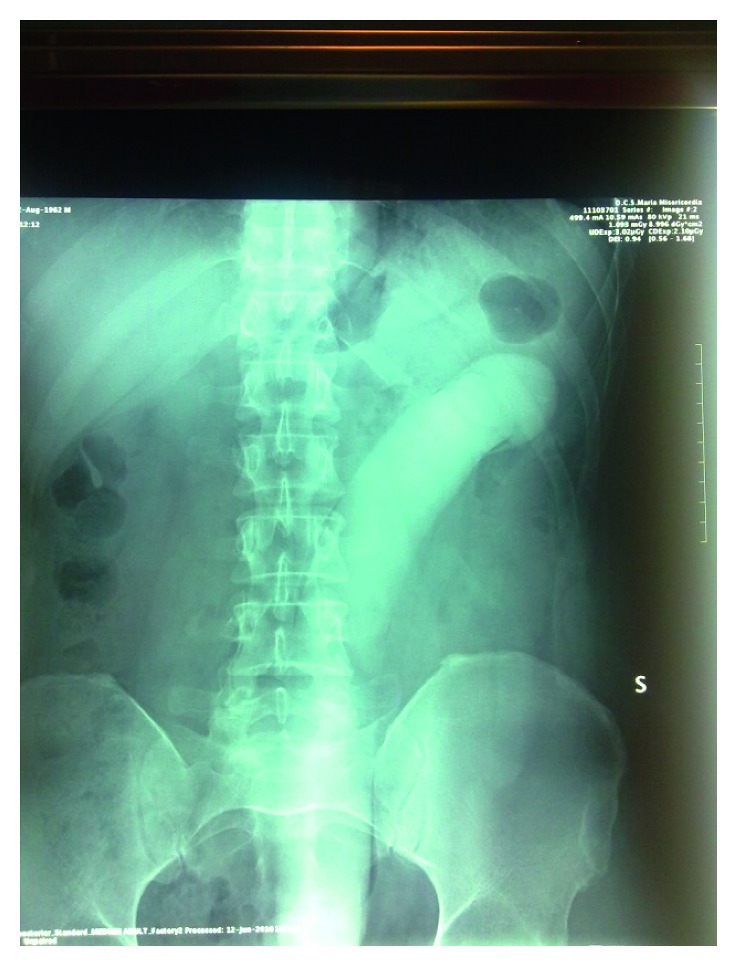
Abdominal X-ray showing the FB reaching the splenic flexure.

In June 2010, a 48-years-old male patient arrived at the Emergency Department (ED) with a self-inserted FB in the rectum that he tried to remove without success. The patient had no symptoms, apart from a mild discomfort. Physical exploration revealed no signs of peritonism and a slight tenderness at palpation of the abdomen. Rectal exploration demonstrated the FB 6 cm proximally from the anal verge. An abdominal X-ray was performed, giving details about object's characteristics: a long silicone penis, reaching the splenic flexure (Figure 1).

**Figure 2:**
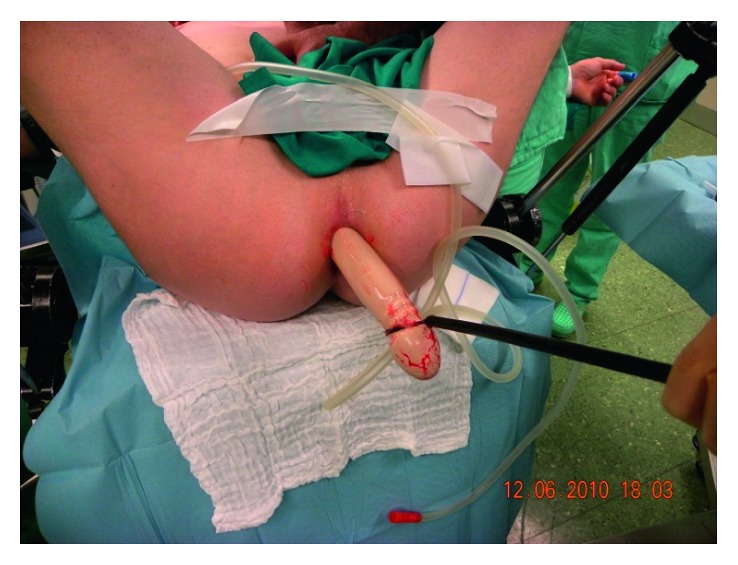
Removal of the FB with Endobag.

No signs of perforation were identified. Laboratory exams were in range. The patient underwent a colonoscopy, in order to remove the object, but every attempt failed. The maximum diameter of endoscopic loop available in our institute was 32 mm. The endoscopist tried to catch the object with this instrument, but it resulted unsuccessful due to the large diameter of the object. Moreover, its slipping surface and the elastic and smooth consistence prevented from catching it with a grasp. The instrument cannot get over the frictional resistance of the FB due to its length, and the endoscopist succeeded in tearing out just a little piece of it. Besides its diameter, the endoscopic loop resulted too flexible to reach our purpose. The patient was therefore transferred to our service and sent to the operating room (OR). Under deep sedation, many unsuccessful attempts to remove the FB with round and Kocher clamps were made. We decided to try the extraction with Endobag, a well-known laparoscopic surgical device. We first removed the plastic bag from the device, using the metallic ring as a noose, particularly useful for the shape of the object, composed by a larger part at both extremities. The metallic ring diameter after removing the plastic bag was 60 mm. After catching the object transanally with the noose on finger guide, we successfully pulled the object out (Figure 2). It resulted to be 400 mm long and 44 mm in diameter. The patient had a regular postoperative course and was dismissed on first postoperative day. No follow-up was performed.

### 2.2. Case 2

In June 2011, a 66-years-old male patient came at the ED with a self-inserted FB in the rectum that he tried to remove without success. The patient had no symptoms. The rectal examination showed the presence of the FB 5 cm proximally from the anal verge. Every attempt of removing the FB using a forceps by the transanal way failed. An abdominal X-ray was performed, showing only the cranial portion of the FB in the rectum; no sign of perforation was identified. After the unsuccessful attempt of the endoscopist, the patient was sent to the operating room. Under deep sedation, we removed the FB using Endobag. We performed the extraction in the same way described in the abovementioned case. The FB was a plastic statuette with a penguin shape 40 mm in diameter. The patient had a regular postoperative course and was dismissed on first postoperative day. No follow-up was performed.

## 3. Discussion

Extraction of a rectal FB can be very challenging for the surgeon. In a series of 87 patients referring to the ED with a rectal FB, 23 (26%) needed to access the OR for removal, and 8 required a laparotomy, while 15 managed to remove the object with an exploration under anesthesia [8]. On the other hand, when attempted in the Emergency Room, extraction had success only in 16% of cases. For this reason, some authors think that patients with a rectal FB should be referred to the surgeon for removal after confirming the presence of the object [9]. The extraction in the OR can be very difficult: many procedures can be used (simple digital manipulation, grasping with forceps, enema, and use of Foley catheter), but, not rarely, many of them result in unable to reach the goal, due to the physical characteristics of the FB that does not permit the application of a single protocol. In our cases, the endoscopist did not succeed in removing the object because of the size (too big to be grasped with the diathermic snare) and the material (too smooth and slipping for the clamp). Therefore, many times the surgeon has to be imaginative and find the most proper instrument for the purpose. Other authors report how they removed FB using perianal anesthetic block, electromagnets, silicone ventouse, or Foley catheters, in relation to shape and size of the object [1, 10, 11]. In our case, the shape of the object, presenting a sort of “head” at the two extremities, was particularly suitable to be grasped with a noose. It was also important to perform the extraction under deep sedation, in order to relax the muscles and prevent contractions due to pain.

## 4. Conclusions

The use of Endobag seems to be a good and effective way to remove FBs from rectum, especially if long shaped. Nevertheless, it is not a simple technique because it cannot be performed during endoscopy, but only on finger guide, and the instrument can only reach FB if close to the anal verge because of the high risk of perforation if inserted in sigmoid colon or more proximally. Hence, we suggest not to go further than 6–8 cm from the anal verge. On the other hand, it should be taken into account because the use of this device can avoid a laparotomy when other known procedures fail.
